# Acetate of Morphia in Burns

**Published:** 1837

**Authors:** T. W. Major

**Affiliations:** Paris, Ky.


### Art. IV.—Acetate of Morphia in Burns.

*Cases of Burns and Scalds successfully treated with the Acetate of Morphia.* By Dr. T. W. Major, of Paris, Ky.

Case 1. In the fall of 1834, I was hastily called to three individuals who had been injured by an accidental explosion of powder in a quarry. Two of them were but slightly affected, and only required the extraction of several fragments of rock, which had found their way into the subcutaneous cellular tissue.

The third was more seriously injured. The hand of the left side was not only dreadfully mangled, but the sclerotic membrane of the eye was so lacerated as to allow the humors to escape. After dressing the wounds thus inflicted, I discovered that my patient had been badly burnt from the wrist up to the origin of the deltoid muscle, the cuticle being entirely removed; and that the pain, thereby induced, together with that from the wound of the hand, was of such a character as to threaten an attack of tetanus. Guided by the belief, that if I could allay the pain in the extremity, and subdue the general irritability of the system, I should be able to prevent the dreadful symptoms with which he was threatened, I immediately prepared a plaster of basilicon, and sprinkling over it three or four grains of the acetate of morphia, applied it from the shoulder to the wrist. The result was, that my patient fell into a quiet sleep, from which he awoke free from pain of every description. When I called the next day and removed the plaster from the arm, I found that the whole surface which had been denuded of its cuticle, was in a state of suppuration; it was washed in tepid milk and water, and the basilicon and morphia reapplied. I prescribed an aperient for the bowels ,and the dressings on the hand were not removed. When I visited the patient again on the succeeding day, which was the second after the accident occurred, I discovered that a nice and delicate cuticle had formed upon the whole surface from the shoulder to the hand, and that no farther treatment was necessary. The wound on the hand and in the eye had yielded to the ordinary remedies in a few days.

Case 2. In November of the same year, Mr. —— requested me to see his little son, who had been scalded on the foot ten days previous to my seeing him. I dressed the foot with basilicon and acetate of morphia, and requested that it should remain undisturbed until I called the next day. Upon my return, the mother expressed hereself much pleased at the salutary effects of the remedy, and said, that she herself was indebted to the composing influence of the morphia upon her little son, for the first night's rest she had had, since the accident occurred. On removing the dressings, the sore was found to be suppurating and void of pain, I directed a tepid wash, as in the former case, and applied the basilicon and morphia during this day and the day succeeding; after which I was not able to see the child, in consequence of its being carried several miles into the country; but the father who returned to town a few days afterwards, assured me that the sore had continued to heal rapidly from the time I last saw it and was well.

Case 3. Mr. McDowell of this vicinity, had a daughter æt. 6 years, whose clothes took fire and were literally consumed upon her. When I reached her father's residence, she was much exhausted, seemed to be in the greatest agony, and unable to contain herself in any position. The cuticle on the left leg was off, from the ancle up as high as the ilium, and on the right side, embracing the space from the glutei muscles below, to the origin of the trapezius above, and extending from thence over the deltoid, and the muscles of the arm down to the elbow. On other portions of the body, the surface was highly inflamed, and the cuticle elevated in many points; indeed there was no portion on the posterior surface of the body unaffected; from head to foot, there was a continuous and dangerously inflamed condition of the skin. I immediately prepared several plasters of basilicon dusted over with morphia, and applied them over the body and extremities of the little girl; and before I finished the application she had fallen into a composed and quiet sleep, which lasted well on to eight hours, and when she awoke, she was entirely free from pain. I used in this first application, not less than five or six grains of morphia, and on my return on the succeeding day, found that where the cuticle had been removed or elevated, the surface below was in a state of suppuration, and that in those points where the cuticle adhered, the inflammation was greatly ameliorated. After using tepid ablutions, for the purpose of cleansing the sores, I applied the basilicon and morphia, as I had done on the previous day, taking care to diminish the quantity of morphia to about two grains. From this time on, my patient mended rapidly.

Case 4. During the present month, November, 1836, I was requested by Mr. J. Hughart of this place, to prescribe for a servant of his, who received a scald while working in a steam factory. 'The cuticle was entirely removed on the ulnar side of the fore arm, from the elbow down, and in some places the cutis vera also, I applied the basilicon and morphia, for four day successively, and discharged him cured.

It is the opinion of many pathologists, that burns are dangerous in proportion to the extent of surface implicated, or in other words, that a deep seated burn is comparatively void of danger, whereas an extensive superficial burn is apt to be followed by a state of morbid excitement, highly prejudicial and dangerous. Impressed with these pathological views with regard to burns, when called to case No. 3, I entertained an opinion, that from the extent of surface implicated, a general consecutive inflammation would inevitably ensue, and ultimately terminate in the death of my patient. But the result of the case as given above, shows, satisfactorily, that the timely application of the remedy in question, will cure; and at the same time prevent the consequential symptoms that may always be apprehended, and which, if they show themselves at all, may lead to a fatal termination.

*November*, 1836.

